# Translation, adaptation, and reliability of a Social Frailty Scale for the Brazilian context: a methodological study

**DOI:** 10.1590/1516-3180.2022.0020.07042022

**Published:** 2022-08-29

**Authors:** Vanessa Almeida Maia Damasceno, Marisa Silvana Zazzetta, Fabiana de Souza Orlandi

**Affiliations:** I PT, MSc. Physiotherapist and Doctoral Student, Postgraduate Program in Nursing, Universidade Federal de São Carlos (UFSCar), São Carlos (SP), Brazil.; II MSc, PhD. Social Worker and Professor, Department of Gerontology, Universidade Federal de São Carlos (UFSCar), São Carlos (SP), Brazil.; III MSc, PhD. Nurse and Professor, Department of Gerontology, Universidade Federal de São Carlos (UFSCar), São Carlos (SP), Brazil.

**Keywords:** Brazil, Social support, Frailty, Aged, Transcultural study, Social frailty, Loneliness, Family relationships

## Abstract

**BACKGROUND:**

Frailty comprises three dimensions: physical, psychological, and social. It is established that social frailty is correlated with several variables, such as quality of life, depression, and loneliness. These findings reinforce the need to investigate and define predictors of social frailty.

**OBJECTIVE:**

To translate, culturally adapt, and assess the reliability of the HALFT scale for Brazil.

**DESIGN AND SETTING:**

Methodological study conducted at Universidade Federal de São Carlos.

**METHODS:**

This study aimed to translate and culturally adapt the HALFT scale from English to Brazilian Portuguese, for which the steps of translation, synthesis of translations, back translation, evaluation by an expert committee, pre-test, and test-retest were followed.

**RESULTS:**

Two independent translators translated the HALFT. The consensual version was established by merging the translations, which were back translated into English by a third translator. The expert committee comprised seven health professionals working in frailty and/or social fields of study. Only one item on the scale had a content validity index of less than one (0.85). The instrument was pre-tested with 35 older adults who considered it clear and understandable, with no suggestion of changes. The reliability analysis (reproducibility) of the adapted version of the HALFT with test-retest of the scale with 23 participants showed a Kappa index of 0.62, showing good agreement.

**CONCLUSION:**

The HALFT scale is translated and adapted for Brazil, and shows good reliability. However, it is necessary to conduct psychometric analysis of the instrument to provide normative data for this population.

## INTRODUCTION

Frailty is increasingly standing out nationally and internationally, as a public health problem, is a predictor of multiple health complications, and tends to increase with aging populations.^1,2^

The definition of frailty has been constantly revised in the literature. However, at its core, the concept of frailty is guided by three pillars. The first is its multidimensionality, which involves both physical and psychosocial factors. The second pillar refers to advancing age; that is, frailty is related to the aging process. Finally, frailty is mutable; therefore, people with frailty can progress or regress according to the mechanisms involved in health care and attention.^2^

Frailty has been considered a one-dimensional syndrome for many years, the impairment thought to be only physical. However, gradual progress in research has made it clear that frailty comprises three dimensions: the physical, psychological, and social.^3^ In this context, social frailty is yet to be defined, since there is no consensus on its criteria.^4^ In addition, scholars indicate that social frailty is a little-explored concept and define it as a continuous risk and/or loss of resources that are important for the fulfillment of one or more basic social needs during life.^5^

Thus, the social environment and social activities developed, besides the physical aspects inherent to frailty, are important factors to be considered in preventing frailty and improving conditions among older adults in the community. Furthermore, a comprehensive understanding of social frailty is suggested, which includes domains of economic status, social networks, and social activities.^6^ It is important to emphasize that frailty carries an individual burden for those who live with it, including impaired quality of life and loneliness.

In this context, Bessa analyzed the social components related to frailty, which showed the different social conditions along the course of life of an individual and the social aspects at each stage that were linked to frailty.^7^ There is evidence in the literature that the accumulation of social deficits is correlated with health risks, which implies that social frailty, when properly assessed, can predict mortality risk in the same way that aspects of physical frailty can.^7^ Additionally, social frailty is correlated with several variables, such as poorer quality of life, depression, and loneliness. These findings reinforce the need to investigate and define the predictors of social frailty.^7,8^

Thus, scholars have developed a screening tool to measure social frailty called the “HALFT scale”. The HALFT is an acronym for Help, Participation, Loneliness, Financial, and Talk, which respectively correspond to the following five items: the inability to help others, limited social participation, loneliness, financial difficulty, and not having anyone to talk to.^8^

There are few global studies on social frailty compared to the other dimensions, and a search for related measures shows that they are nonexistent in Brazil. Therefore, a Brazilian version of the HALFT scale for rapid screening of social frailty in older Brazilians is extremely relevant.

## OBJECTIVE

Translate, culturally adapt and assess the reliability of the HALFT scale for Brazil.

## METHODS

### Study type

**Figure 1:**
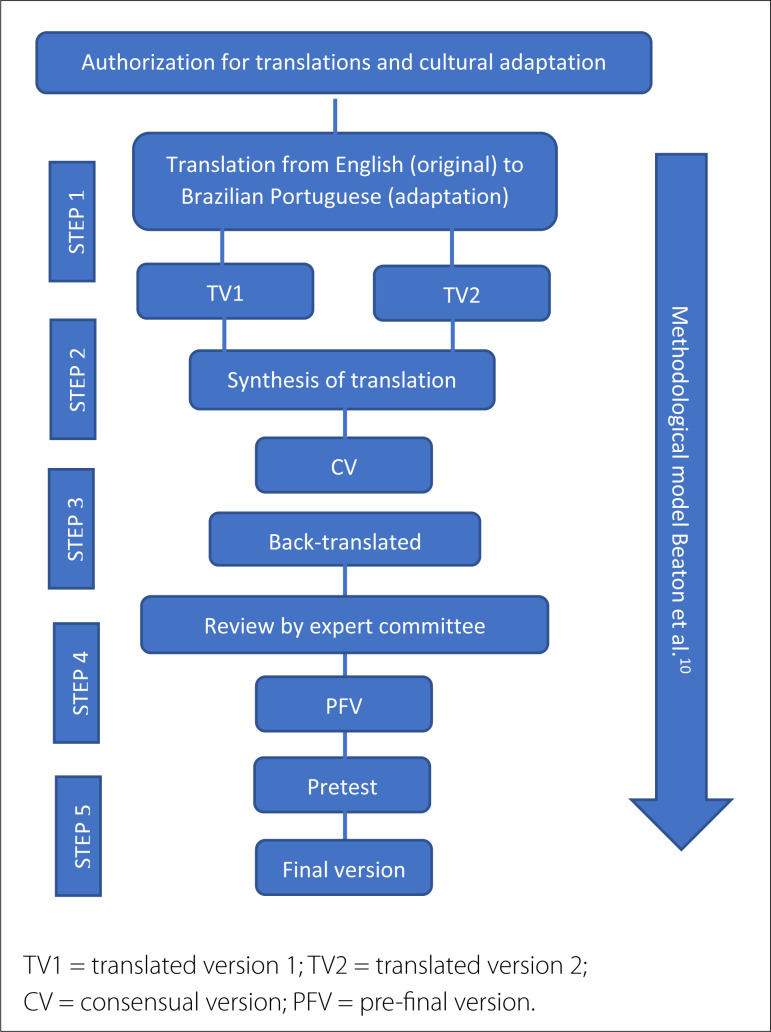
Scheme of the methodological model and phases adopted in the translation and cultural adaptation process of the HALFT scale.

Source: Figure adapted.^10^

This is a methodological study^9^ proposing the translation and cultural adaptation of the HALFT scale from English to Brazilian Portuguese. This methodology requires a prior design of all steps to be developed^10^ (Figure 1). This process of translation and cultural adaptation is conducted so that there is equivalence between the tool in its source language and target language.^11^

### Sample

Individuals aged 60 years and above who have a minimum level of literacy that enables the reading and interpretation of the evaluation questions, participated in the present study. Persons who had severe vision impairment and did not have access to the internet or digital research platforms were not included. All participants signed a Free and Informed Consent Form, and the study was approved on December 19, 2020, by the Ethics Committee on Human Experimentation of the Universidade Federal de São Carlos (UFScar) (4.477.339).

### Questionnaire structure

The HALFT scale comprises five items: the inability to help others, limited social participation, loneliness, financial difficulty, and not having anyone to talk to.^8^

The item regarding the ability to help others was measured by asking participants if they had been able to help their friends or family in the past 12 months. Limited social participation was assessed by asking participants if they had engaged in any social or leisure activities in the previous 12 months. Loneliness was defined as feeling lonely during the last week. Financial difficulty was determined by asking participants if their income had been sufficient to live on for the past 12 months. The final item verified if the participants had someone they could talk to every day.

The score of the HALFT scale ranges from 0 to 5 points, with a score of zero being considered not socially frail, 1 or 2 as pre-socially frail, and a score of ≥ 3 indicating socially frailty.^8^

### Translation into Portuguese and cross-cultural adaptation

The instrument was formally authorized by the authors of the original scale for the creation of a Brazilian version. The researchers strictly followed scientific and ethical guidelines and the research was approved by the Human Research Ethics Committee of Universidade Federal de São Carlos and fully abided by the recommendations of Resolution 466/2012 of the Brazilian National Health Council (Conselho Nacional de Saúde, CNS) of the Ministry of Health.

As mentioned above, this type of study is conducted in steps. The first was the translation of the original English version into Brazilian Portuguese, which was conducted by two independent, qualified translators with fluency in Brazilian Portuguese. It is noteworthy that both were experienced with this type of translation and familiar with health terminology. This translation step produced the translated version 1 (TV1) and translated version 2 (TV2).

The synthesis of the translations (TV1 and TV2) was conducted in the second step, thus constituting the consensual version. This process was conducted jointly by the researchers and translators. The subsequent step of back-translation utilized a third translator whose native language was English and who fulfilled the same requirements as the others but lacked prior knowledge of the instrument. Once the back-translated version (BTV) was ready, the instrument was sent to the authors for analysis and approved.

Cultural adaptation was performed as the fourth step, which included a content assessment and cultural equivalence analysis. A committee of experts comprising seven members was formed, all with PhDs, working in the field of research, with expertise in frailty and/or social aspects, and fluency in English. Upon agreeing to be part of the committee, they received an explanatory letter about the instrument clarifying the requested analysis. The committee members were asked to analyze the four versions of the instrument (original, translation 1, translation 2, and consensual version) to establish the content validity index, which aimed to verify the concordance of the judges. For this analysis, a four-point Likert scale was used, in which items with responses corresponding to a score of 1 and/or 2 were to be revised or excluded, and items with a score of 3 and/or 4 were to be calculated. Based on the sum of the responses of each judge on each item divided by the total number of responses, a concordance value was calculated, with a recommended result of 0.78 and over^12^ to confirm the equivalence of the instrument after the entire process. The pre-final version (PFV) of the HALFT scale was obtained after a thorough evaluation by the experts.

In the fifth and last step, the instrument's PFV was pretested with an older adult sample, with characteristics as previously described. As this was a period of social distancing due to the pandemic, we decided to deliver the instrument to the participants and collect it 10 days later. Three instruments were delivered to the participants: a caregiver characterization questionnaire (sociodemographic information); the pre-final version of the HALFT with two extra columns that checked the clarity of the terms of each question and asked the participants for possible suggestions and adjustments to the questions; a general Disabkids questionnaire to assess the clarity of the instrument to be adapted. The HALFT scale was finalized at the end of this step.

### Evaluation of the reliability of the HALFT scale

The HALFT scale was applied to 23 older adult participants twice with an interval of 15 days to assess its reliability (reproducibility). The research was conducted digitally, that is, with the scale typed in an online format. Upon contact, participants received instructions on how to proceed with the evaluation. Fifteen days later, the evaluator contacted them again to forward the instrument and request that they respond a second time.

### Statistical analysis

Excel for Windows was used to tabulate the data, which were processed using the IBM SPSS statistics, version 20.0 (IBM Corporation, Armonk, New York, United States), in which a descriptive analysis of the sociodemographic characteristics of the participants was performed. The content validity index (CVI) of the HALFT scale items was calculated using the criteria proposed by Lynn in 1986, with an expected value greater than 0.78.^12^ The Kappa statistic was used for reliability analysis. The criteria proposed by Landis and Koch^13^ were followed. A 95% confidence interval (CI) was adopted.

## RESULTS

After the initial translation process, the statistical analysis was conducted to reach the final version of the instrument and assess its reliability.

We began the analyses by checking the content validity of the scale approved by the expert committee. The CVI of the five questions of the instrument was calculated and only the first question had a value of 0.85 and the rest a value of 1, indicating satisfactory content validity (Table 1).

**Table 1:** Content validity index of the HALFT Scale items as evaluated by the Expert Committee

Items	Judge 1	Judge 2	Judge 3	Judge 4	Judge 5	Judge 6	Judge 7	Content validity index
1	4	3	3	4	2	4	4	0.85
2	4	3	4	4	4	4	4	1.00
3	3	3	4	4	4	4	4	1.00
4	4	4	4	4	4	4	4	1.00
5	4	3	4	4	4	4	4	1.00

After the expert committee's analysis, a pre-test was conducted. For this step, 35 older adults participated, of whom 60% were female and 65.71% were married or had a common-law marriage (Table 2). Their average monthly income was R$ 3,014.28 (standard deviation, SD: 3,789.51) and the average amount of medication in terms of the number of pills taken per day was 3.80 (SD: 3.38).

**Table 2:** Descriptive statistics of the sociodemographic characterization of pre-test participants

Variable	Category	Frequency	%
**Gender**	Female	21	60
	Male	14	40
**Marital status**	Single	1	2.81
	Married	23	65.71
	Widow/er	5	14.29
	Divorced	6	17.14
**Education**	Elementary school	7	20
	Middle school	8	22.86
	High school	9	25.71
	Higher education	11	31.43
**Do you have any disease diagnosed?**	Yes	27	77.14
	No	8	22.86

When applying the PFV of the scale, all participants rated the five items of the instrument as clear and did not suggest any changes. The scale's relevance, as well as the clarity of the items in the PFV, was assessed using the general clarity questionnaire adapted from Disabkids, and showed that the participants considered the instrument to be very good (n = 35; 100%).

Even though the consensual version analyzed by the judges and participants obtained a good evaluation, they suggested adjusting the questions so that they all started with the time frame, thus facilitating the interpretation by the participants (Table 3).

**Table 3:** Readjustment of items from the consensual version to the final version

Item	Consensual version	Final version
**1**	Have you helped a friend or family member in the past 12 months?	In the last 12 months, have you helped friends and/or family members in any kind of need?
**2**	Have you participated in any social or leisure activities in the last 12 months?	In the last 12 months, have you participated and/or been involved in any social or leisure activities?
**3**	Have you felt lonely in the last 12 months?	In the last week, have you felt lonely?
**4**	Was your income enough to live on for the last 12 months?	Do you think your income was enough to live on in the last 12 months?
**5**	Do you have someone to talk to daily?	Do you have someone you can talk to every day?

To establish the reliability of the HALFT scale, we analyzed the responses of 23 older adult participants, of which 60.87% were female, and 47.83% had completed higher education. Their average monthly income was R$ 4,400.00 (SD: 4,443.89) and the average amount of medication taken daily in pill form was 5.39 (SD: 3.8) (Table 4).

**Table 4:** Descriptive statistics of the sociodemographic characterization of test-retest participants

Variable	Category	Frequency	%
**Gender**	Female	14	60.87
	Male	9	39.13
**Marital Status**	Married	15	65.22
	Widow/er	7	30.43
	Divorced	1	4.35
**Education**	Elementary school	7	30.43
	Middle school	2	8.70
	High school	5	21.74
	Higher education	9	39.13
**Do you have any disease diagnosed?**	Yes	15	65.22
	No	8	34.78
**Do you take medication on an ongoing basis?**	Yes	21	91.3
	No	2	8.7

Reproducibility analysis was performed using weighted Kappa, with a value of 0.62, showing good concordance according to the reference adopted (Table 5).^13^

**Table 5:** Reproducibility of the HALFT scale (final version)

Item	Test		Retest		Weighted Kappa (95% CI)
	0	1	0	1	
1	19 (82.61%)	4 (17.39%)	20 (86.96%)	3 (13.04%)	
2	8 (34.78%)	15 (65.22%)	9 (39.13%)	14 (60.87%)	
3	20 (86.96%)	3 (13.04%)	20 (86.96%)	3 (13.04%)	0.62 (0.37–0.87)
4	16 (69.57%)	7 (30.43%)	18 (78.26%)	5 (21.74%)	
5	23 (100.00%)	0 (0.00%)	21 (91.30%)	2 (8.70%)	

CI = confidence interval.

## DISCUSSION

Recently, much progress has been made in understanding frailty. However, there are still many gaps, from arriving at a consensus on the definition of frailty and associated variables to identifying the means of assessment. Scholars have emphasized the importance of conducting clinical trials for the development of preventive and management strategies for this syndrome.^2^

Frailty is a challenge for public health, as it is related to functional decline, depressive symptoms, and social isolation, in addition to being a predictor of mortality. It is believed that older adults can provide information on their experience that can help us design better health systems and social assistance to meet this demand for care, thus avoiding further harm to health.^14,15^

National and international studies have shown the need to provide the Brazilian population with an instrument for tracking social frailty, given the scarcity of studies and the need for acknowledgment of this syndrome, thus enabling the development of strategies and resources to prevent and address it. Bunt et al. emphasized in their scoping review on social frailty in older adults that it is the least explored variable when compared to physical and cognitive frailty.^5^ The authors also reveal that older adults increasingly require assistance for social issues, whether through social relationships or even environments, which is why scholars stress the need for concrete conceptualization and effective assessments for this variable.

Faced with this need, Ma, Sun, and Tang^8^ developed the HALFT Scale to screen for social frailty by applying it to 1,697 older adults living in the community in the city of Beijing. After evaluation, the authors reported that the HALFT scale was associated with adverse health, as well as social outcomes, indicating its usefulness. However, they also report the need to continue with psychometric evaluations through further studies in order to determine the efficacy of the scale.^8^ Hence, the decision to conduct this process for the future availability of the scale in Brazil.

The availability of the HALFT scale in Brazil will enable research contributing to the advancement of scientific knowledge in the country on social frailty and the potential for comparisons with global findings. In addition, it will also allow health professionals who assist the aging population of Brazil in different health and social contexts to be equipped with a rapid social frailty screening scale, allowing them to identify individuals in the progression of social frailty, early. This will give them a chance to intervene and improve this domain of frailty, also impacting the physical and cognitive dimensions, as seen in the aforementioned international studies.

It is known that methodological studies have steps to be followed.^16^ In Brazil, the number of studies of this type has increased because it is extremely important to have an instrument that is adapted and validated for local populations.^17^

The present study strictly followed all steps recommended by Beaton et al.^10^ The initial step was conducted by two translators, both of English nationality and fluent in Brazilian Portuguese. According to the literature, it is recommended that the translation step be undertaken by fluent, independent translators with specialist qualifications to achieve efficiency in this process.^18^

The second step included the synthesis of the translations by the researchers and the formation of the consensual version. In this study, there was no discrepancy between the translations. A few differences in words but with the same meanings were present, in which case, the more commonly used word was chosen. To resolve differences and ambiguities, the translations were read, and a consensual version^18^ was prepared. Subsequently, back-translation to the original language was performed to verify whether the instrument, after the translation and synthesis processes, remained faithful to the original, which was confirmed in the present study.

In the present study, an expert committee evaluated the instrument, as described in the Methods section. The five items of the scale were individually evaluated, in a process that ensured that the PFV was clear and understandable to the new culture. The purpose of IVC is to verify the committee's concordance on the scale's questions, that is, the equivalence of the questions in the different versions.^19^

Once the PFV was ready, a pre-test was conducted. Its objective was to apply the scale to a small sample and to verify whether the participants encountered any difficulties related to the understanding and clarity of the instrument's questions.^20^ In the present study, participants did not report difficulties and did not suggest changes, so the final version of the HALFT scale was obtained.

The last step of the present study was to verify the reliability of the final version of the scale using the test-retest method, which involved the participants responding to the scale at two time points with a 15-day gap, as recommended in the literature.^21^ Reliability, according to the Consensus-based Standards for the Selection of Health Measurement Instruments (COSMIN), is the degree to which an instrument is free from measurement error.^22^ The present study used the weighted Kappa and 95% CI were estimated^13^ and observed with the analysis values of 0.62, which shows good reliability.

As a limitation, we can highlight the scarcity of studies on this subject, making it difficult to deepen the discussion. Another limitation of the present study is that data were collected through a digital platform which excluded people from participating because they did not have access to the digital environment or because they were unaware of the study. However, it is important to emphasize that this study stands out for being innovative and aiming to provide an instrument for assessing social frailty, serving as an addition to the limited supply of relevant and current studies required to remedy this gap regarding the construct of frailty.

## CONCLUSION

Based on the objective of the study and the results obtained, it is concluded that the HALFT scale is properly translated and adapted to Brazil, and shows good reliability when it is self-applied to older Brazilian people in the community.
